# Supplementary figures and images for: Derivation and validation of a clinical severity score for acutely ill adults with suspected COVID-19: The PRIEST observational cohort study
Source: PLoS One. 2021 Jan 22;16(1):e0245840. doi: 10.1371/journal.pone.0245840 (PMC7822515; doi:10.1371/journal.pone.0245840)

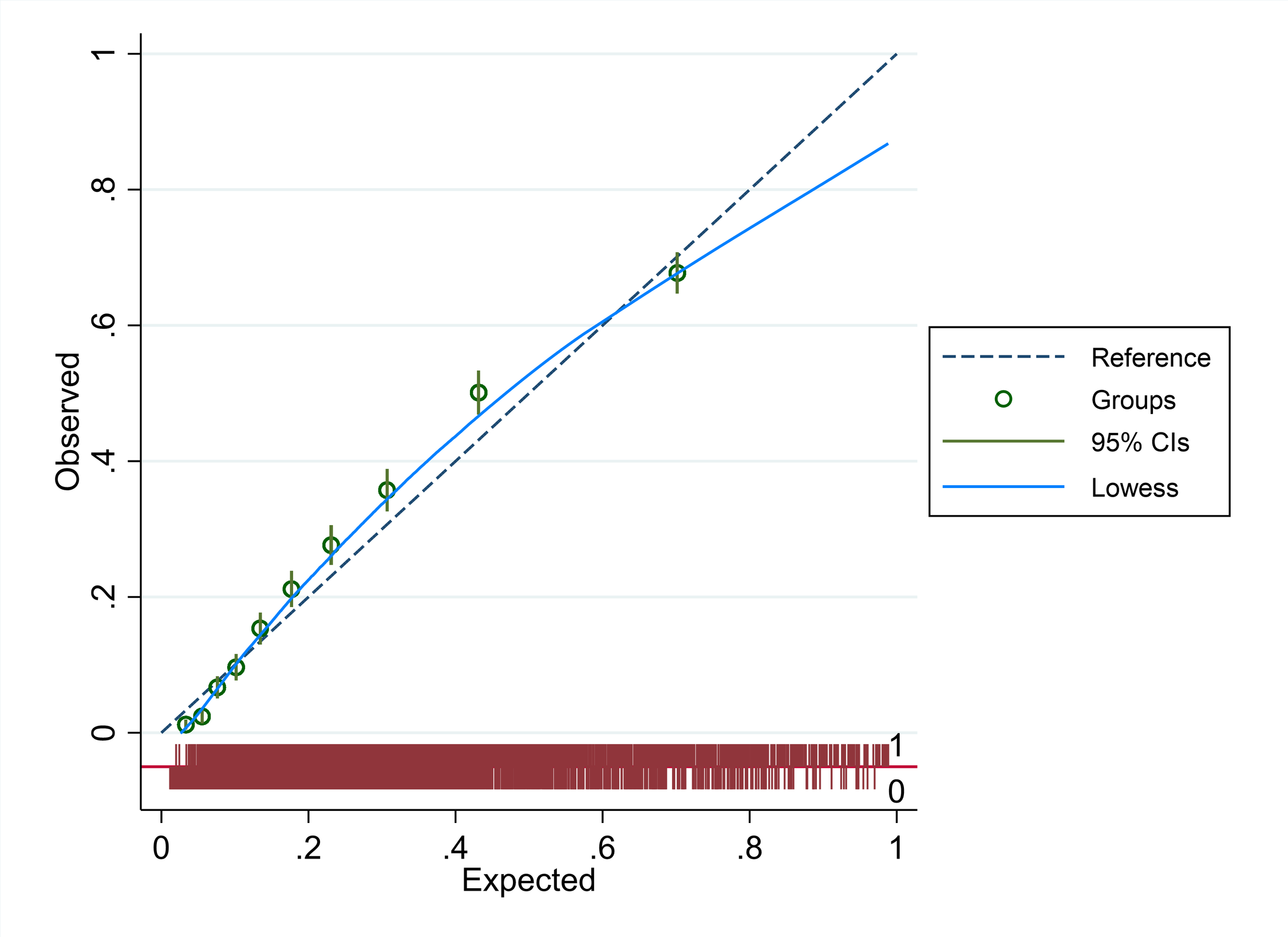

Supplement: S1 Fig — (TIF) [file pone.0245840.s001.tif]

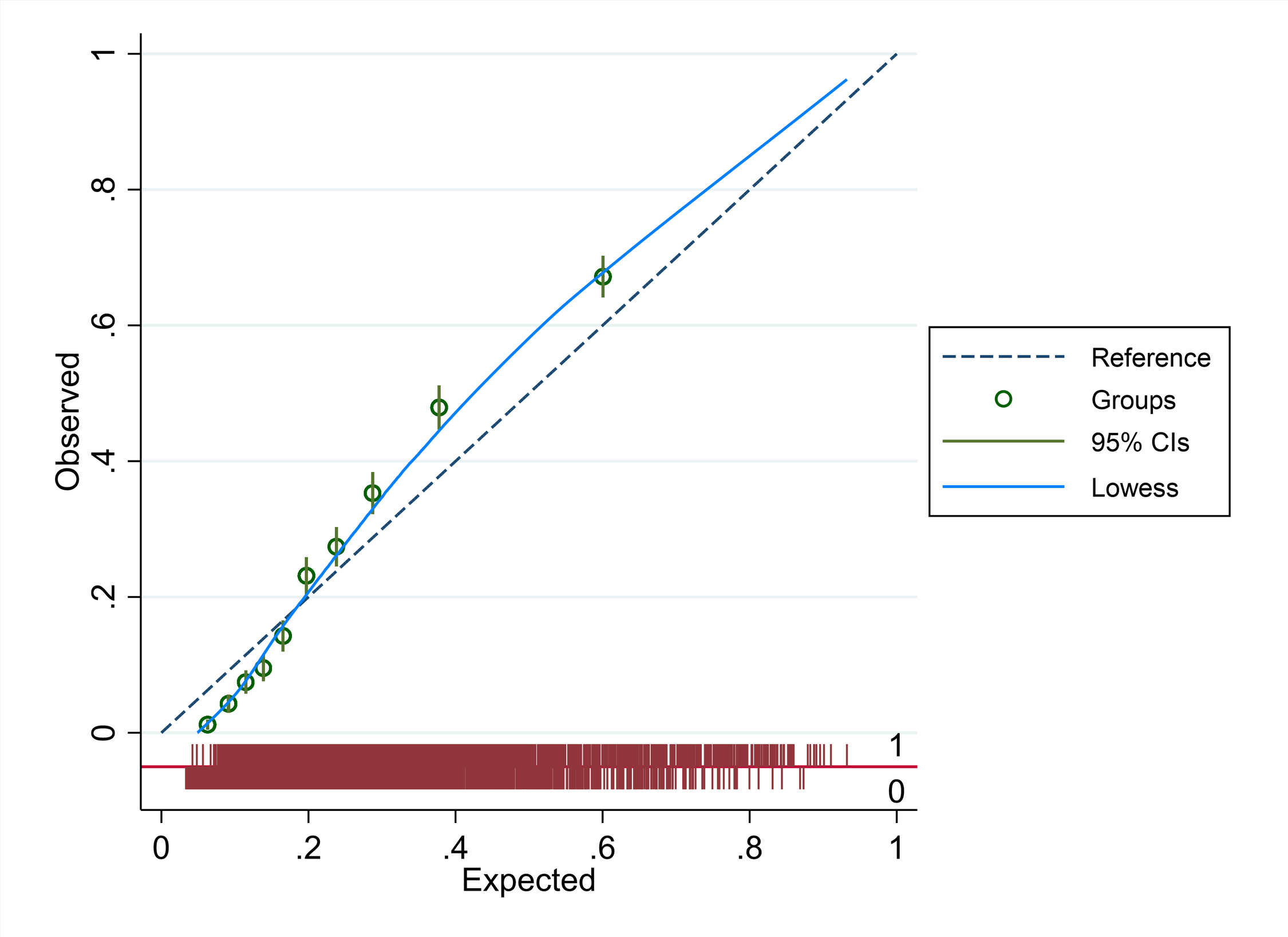

Supplement: S2 Fig — (TIF) [file pone.0245840.s002.tif]

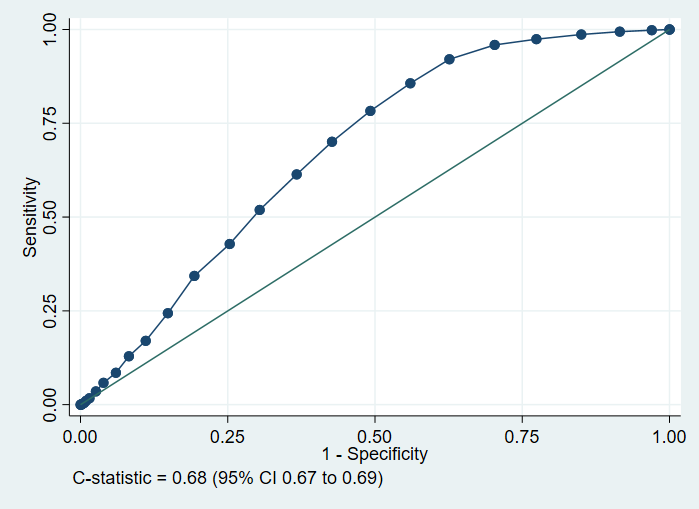

Supplement: S3 Fig — (TIF) [file pone.0245840.s003.tif]

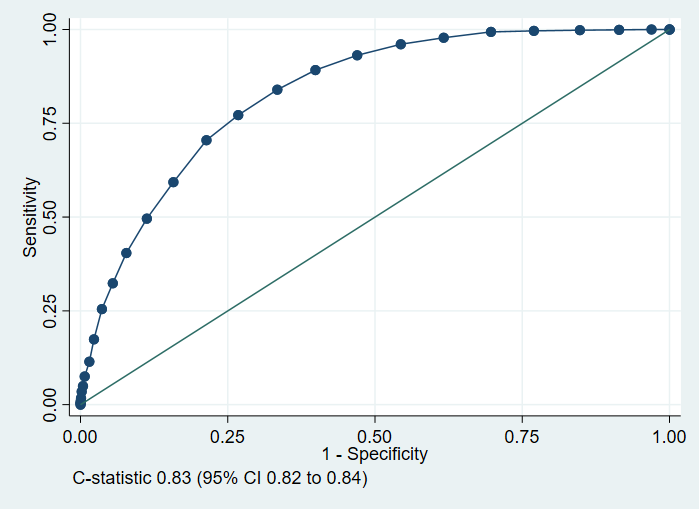

Supplement: S4 Fig — (TIF) [file pone.0245840.s004.tif]
