# Supplementary material for: Derivation and validation of a clinical severity score for acutely ill adults with suspected COVID-19: The PRIEST observational cohort study
Source: PLoS One. 2021 Jan 22;16(1):e0245840. doi: 10.1371/journal.pone.0245840 (PMC7822515; doi:10.1371/journal.pone.0245840)
Supplement: S1 Table — (DOCX) [file pone.0245840.s005.docx]

### S1 Table: Multivariable analysis, complete case (N=5988)

| **Lasso variable selection (unrestricted)** | | |  | **Lasso variable selection (restricted to 10)** | | |
| --- | --- | --- | --- | --- | --- | --- |
| C-statistic: 0.83 (95% CI 0.82 to 0.84) | | |  | C-statistic: 0.82 | | |
| **Parameter** | **Coefficient** | |  | **Parameter** | **Coefficient** | |
|  | **Unstandardised** | **Standardised** |  |  | **Unstandardised** | **Standardised** |
| Age | 0.019 | 0.373 |  | Age | 0.014 | 0.28 |
| Symptom duration | -0.002 | -0.016 |  | ln(respiratory rate) | 0.895 | 0.239 |
| ln(respiratory rate) | 1.129 | 0.302 |  | Systolic BP^-2 | 4851.16 | 0.122 |
| Heart rate | 0.002 | 0.034 |  | Oxygen saturation/inspired ratio | -0.005 | -0.581 |
| ln(temperature)*temperature^3 | 0 | 0.072 |  | Medication count | 0.004 | 0.018 |
| Systolic BP^-2 | 9458.016 | 0.238 |  | Male sex | 0.023 | 0.012 |
| Oxygen saturation/inspired ratio | -0.006 | -0.617 |  | Renal impairment | 0.037 | 0.011 |
| Medication count | 0.017 | 0.075 |  | Performance status level 1 | -0.082 | -0.041 |
| Male sex | 0.31 | 0.155 |  | Respiratory distress | 0.08 | 0.015 |
| Shortness of breath | 0.112 | 0.049 |  | Consciousness alert | -0.44 | -0.114 |
| Renal impairment | 0.287 | 0.083 |  | Constant | -2.967 | -1.468 |
| Asthma | -0.026 | -0.009 |  |  |  |  |
| Diabetes | 0.137 | 0.056 |  |  |  |  |
| Active malignancy | 0.058 | 0.013 |  |  |  |  |
| Immunosuppression | 0.111 | 0.019 |  |  |  |  |
| Other chronic lung disease | -0.039 | -0.015 |  |  |  |  |
| Hypertension | 0.03 | 0.014 |  |  |  |  |
| Clinically obese | 0.196 | 0.051 |  |  |  |  |
| Tobacco or vape user | -0.202 | -0.061 |  |  |  |  |
| Covid contact | 0.197 | 0.06 |  |  |  |  |
| Performance status |  |  |  |  |  |  |
| 1 | -0.149 | -0.074 |  |  |  |  |
| 2 | -0.187 | -0.061 |  |  |  |  |
| 4 | 0.112 | 0.039 |  |  |  |  |
| 5 | 0.061 | 0.015 |  |  |  |  |
| Respiratory distress | 0.46 | 0.084 |  |  |  |  |
| Respiratory exhaustion | 0.296 | 0.041 |  |  |  |  |
| Dehydration | 0.683 | 0.086 |  |  |  |  |
| Consciousness |  |  |  |  |  |  |
| Alert | -0.537 | -0.138 |  |  |  |  |
| Pain | 0.551 | 0.059 |  |  |  |  |
| Unresponsive | 0.167 | 0.011 |  |  |  |  |
| Constant | -5.446 | -1.623 |  |  |  |  |
