# Supplementary material for: Derivation and validation of a clinical severity score for acutely ill adults with suspected COVID-19: The PRIEST observational cohort study
Source: PLoS One. 2021 Jan 22;16(1):e0245840. doi: 10.1371/journal.pone.0245840 (PMC7822515; doi:10.1371/journal.pone.0245840)
Supplement: S2 Table — (DOCX) [file pone.0245840.s006.docx]

### S2 Table: Multivariable analysis, using multiple imputation (50 imputations; N=11636)

| **Lasso variable selection (unrestricted)** | | | |  | **Lasso variable selection (restricted to 10)** | | | | |
| --- | --- | --- | --- | --- | --- | --- | --- | --- | --- |
| C-statistic: 0.85 (95% CI 0.84 to 0.86) | | | |  | C-statistic: 0.85 (95% CI 0.84 to 0.85) | | | | |
| **Parameter** | **Average coefficient** | | **No. times selected** |  | **Parameter** | **Average coefficient** | | **No. times selected** |  |
|  | **Unstandardised** | **Standardised** |  |  |  | **Unstandardised** | **Standardised** |  |  |
| Age | 0.021 | 0.428 | 50 |  | Age | 0.017 | 0.333 | 50 |  |
| Symptom duration | -0.006 | -0.054 | 50 |  | ln(respiratory rate) | 1.060 | 0.280 | 50 |  |
| ln(respiratory rate) | 1.313 | 0.347 | 50 |  | Systolic BP^-2 | 4400.528 | 0.111 | 50 |  |
| Heart rate | 0.002 | 0.034 | 50 |  | Oxygen saturation/inspired ratio | -0.006 | -0.605 | 50 |  |
| ln(temp)*temp^3 | 0.000 | 0.029 | 50 |  | Male sex | 0.030 | 0.015 | 50 |  |
| Systolic BP^-2 | 8662.087 | 0.219 | 50 |  | Renal impairment | 0.065 | 0.019 | 50 |  |
| Oxygen saturation/inspired ratio | -0.006 | -0.647 | 50 |  | Performance status |  |  |  |  |
| Medication count | 0.008 | 0.036 | 50 |  | 1 | -0.188 | -0.094 | 50 |  |
| Male sex | 0.309 | 0.155 | 50 |  | 4 | 0.045 | 0.015 | 50 |  |
| Shortness of breath | 0.199 | 0.088 | 50 |  | Respiratory distress | 0.154 | 0.026 | 50 |  |
| Previous attendance | 0.057 | 0.017 | 50 |  | Consciousness alert | -0.525 | -0.138 | 50 |  |
| Heart disease | -0.046 | -0.019 | 50 |  |  |  |  |  |  |
| Renal impairment | 0.306 | 0.088 | 50 |  | *Performance status*  *level 5* | *0.021* | *0.005* | *48* |  |
| Asthma | -0.118 | -0.043 | 50 |  | *Medication count* | *0.001* | *0.004* | *8* |  |
| Diabetes | 0.176 | 0.071 | 50 |  | *Diabetes* | *0.005* | *0.002* | *5* |  |
| Active malignancy | 0.294 | 0.064 | 50 |  |  |  |  |  |  |
| Immunosuppression | 0.301 | 0.049 | 50 |  | Constant | -3.477 | -1.598 | 50 |  |
| Other chronic lung disease | -0.088 | -0.034 | 50 |  |  |  |  |  |  |
| Hypertension | 0.071 | 0.033 | 50 |  |  |  |  |  |  |
| Clinically obese | 0.276 | 0.067 | 50 |  |  |  |  |  |  |
| Tobacco or vape user | -0.265 | -0.081 | 50 |  |  |  |  |  |  |
| Covid contact | 0.200 | 0.059 | 50 |  |  |  |  |  |  |
| Performance status |  |  |  |  |  |  |  |  |  |
| 1 | -0.214 | -0.107 | 50 |  |  |  |  |  |  |
| 2 | -0.167 | -0.054 | 50 |  |  |  |  |  |  |
| 4 | 0.226 | 0.077 | 50 |  |  |  |  |  |  |
| 5 | 0.249 | 0.064 | 50 |  |  |  |  |  |  |
| Respiratory distress | 0.488 | 0.082 | 50 |  |  |  |  |  |  |
| Respiratory exhaustion | 0.449 | 0.056 | 50 |  |  |  |  |  |  |
| Dehydration | 0.490 | 0.057 | 50 |  |  |  |  |  |  |
| Consciousness |  |  |  |  |  |  |  |  |  |
| Alert | -0.630 | -0.165 | 50 |  |  |  |  |  |  |
| Pain | 0.312 | 0.033 | 50 |  |  |  |  |  |  |
| Unresponsive | 0.424 | 0.033 | 50 |  |  |  |  |  |  |
|  |  |  |  |  |  |  |  |  |  |
| *Cough* | *0.014* | *0.007* | *38* |  |  |  |  |  |  |
| *Pregnant* | *-0.059* | *-0.004* | *11* |  |  |  |  |  |  |
| *Steroid use* | *-0.019* | *-0.003* | *6* |  |  |  |  |  |  |
| *Fever* | *-0.011* | *-0.005* | *3* |  |  |  |  |  |  |
|  |  |  |  |  |  |  |  |  |  |
| Constant | -5.596 | -1.777 | 50 |  |  |  |  |  |  |

|  |  |
| --- | --- |
