# Supplementary material for: Derivation and validation of a clinical severity score for acutely ill adults with suspected COVID-19: The PRIEST observational cohort study
Source: PLoS One. 2021 Jan 22;16(1):e0245840. doi: 10.1371/journal.pone.0245840 (PMC7822515; doi:10.1371/journal.pone.0245840)
Supplement: S3 Table — (DOCX) [file pone.0245840.s007.docx]

### S3 Table: Multivariable analysis, using deterministic imputation (N=9891)

| **Lasso variable selection (unrestricted)**  C-statistic: 0.83 (95% CI 0.82 to 0.84) | | | |  | **Lasso variable selection (restricted to 10)**  C-statistic: 0.82 (95% CI 0.81 to 0.83) | | | |
| --- | --- | --- | --- | --- | --- | --- | --- | --- |
| **Parameter** | **Coefficient** | |  | | **Parameter** | **Coefficient** | |  |
|  | **Unstandardised** | **Standardised** |  | |  | **Unstandardised** | **Standardised** |  |
| Age | 0.023 | 0.45 |  | | Age | 0.017 | 0.349 |  |
| Symptom duration | -0.004 | -0.039 |  | | ln(respiratory rate) | 1.219 | 0.318 |  |
| ln(respiratory rate) | 1.479 | 0.385 |  | | Systolic BP^-2 | 4989.762 | 0.122 |  |
| Heart rate | 0.003 | 0.055 |  | | Oxygen saturation/inspired ratio | -0.005 | -0.481 |  |
| ln(temperature)*temperature^3 | 0 | 0.008 |  | | Male sex | 0.049 | 0.025 |  |
| Systolic BP^-2 | 9572.929 | 0.235 |  | | Renal impairment | 0.107 | 0.03 |  |
| Oxygen saturation/inspired ratio | -0.005 | -0.512 |  | | Performance status |  |  |  |
| Medication count | 0.001 | 0.006 |  | | 1 | -0.132 | -0.066 |  |
| Male sex | 0.329 | 0.164 |  | | 4 | 0.04 | 0.013 |  |
| Shortness of breath | 0.184 | 0.081 |  | | Respiratory distress | 0.041 | 0.007 |  |
| Previous attendance | 0.049 | 0.015 |  | | Consciousness alert | -0.484 | -0.117 |  |
| Heart disease | -0.002 | -0.001 |  | | Constant | -4.222 | -1.599 |  |
| Renal impairment | 0.355 | 0.101 |  | |  |  |  |  |
| Steroid use | 0.066 | 0.01 |  | |  |  |  |  |
| Asthma | -0.136 | -0.05 |  | |  |  |  |  |
| Diabetes | 0.198 | 0.079 |  | |  |  |  |  |
| Active malignancy | 0.317 | 0.067 |  | |  |  |  |  |
| Immunosuppression | 0.269 | 0.044 |  | |  |  |  |  |
| Other chronic lung disease | -0.052 | -0.02 |  | |  |  |  |  |
| Hypertension | 0.069 | 0.032 |  | |  |  |  |  |
| Clinically obese | 0.333 | 0.082 |  | |  |  |  |  |
| Tobacco or vape user | -0.231 | -0.071 |  | |  |  |  |  |
| Covid contact | 0.198 | 0.059 |  | |  |  |  |  |
| Performance status |  |  |  | |  |  |  |  |
| 1 | -0.149 | -0.074 |  | |  |  |  |  |
| 2 | -0.124 | -0.039 |  | |  |  |  |  |
| 4 | 0.227 | 0.074 |  | |  |  |  |  |
| 5 | 0.142 | 0.034 |  | |  |  |  |  |
| Respiratory distress | 0.37 | 0.062 |  | |  |  |  |  |
| Respiratory exhaustion | 0.468 | 0.06 |  | |  |  |  |  |
| Dehydration | 0.441 | 0.05 |  | |  |  |  |  |
| Consciousness |  |  |  | |  |  |  |  |
| Alert | -0.639 | -0.155 |  | |  |  |  |  |
| Pain | 0.475 | 0.047 |  | |  |  |  |  |
| Unresponsive | 0.288 | 0.018 |  | |  |  |  |  |
| Constant | -6.219 | -1.772 |  | |  |  |  |  |
