# Supplementary material for: Derivation and validation of a clinical severity score for acutely ill adults with suspected COVID-19: The PRIEST observational cohort study
Source: PLoS One. 2021 Jan 22;16(1):e0245840. doi: 10.1371/journal.pone.0245840 (PMC7822515; doi:10.1371/journal.pone.0245840)
Supplement: S4 Table — (DOCX) [file pone.0245840.s008.docx]

### S4 Table: Logistic regression model based on selected categorised predictor variables

| Predictor | Score allocated | Coefficient | SE | z | P-value | Upper 95% CI | Lower 95% CI |
| --- | --- | --- | --- | --- | --- | --- | --- |
| Respiratory rate |  |  |  |  |  |  |  |
| 12-20* | 0 |  |  |  |  |  |  |
| 9-11 | 1 | 0.598 | 1.387 | 0.43 | 0.666 | -2.121 | 3.317 |
| 21-24 | 2 | 0.330 | 0.089 | 3.69 | 0 | 0.155 | 0.505 |
| <9 or >24 | 3 | 0.684 | 0.082 | 8.3 | 0 | 0.523 | 0.846 |
|  |  |  |  |  |  |  |  |
| Oxygen saturation |  |  |  |  |  |  |  |
| >95%* | 0 |  |  |  |  |  |  |
| 94-95% | 1 | 0.361 | 0.090 | 4.02 | 0 | 0.185 | 0.537 |
| 92-93% | 2 | 0.697 | 0.112 | 6.24 | 0 | 0.478 | 0.916 |
| <92% | 3 | 1.128 | 0.085 | 13.24 | 0 | 0.961 | 1.295 |
|  |  |  |  |  |  |  |  |
| Heart rate |  |  |  |  |  |  |  |
| 51-90* | 0 |  |  |  |  |  |  |
| 41-50 or 91-110 | 1 | -0.054 | 0.077 | -0.7 | 0.486 | -0.205 | 0.097 |
| 111-130 | 2 | 0.141 | 0.096 | 1.46 | 0.143 | -0.048 | 0.329 |
| <41 or >130 | 3 | 0.295 | 0.141 | 2.09 | 0.037 | 0.018 | 0.572 |
|  |  |  |  |  |  |  |  |
| Systolic BP |  |  |  |  |  |  |  |
| 111-219* | 0 |  |  |  |  |  |  |
| 101-110 | 1 | 0.229 | 0.109 | 2.11 | 0.035 | 0.016 | 0.442 |
| 91-100 | 2 | 0.596 | 0.145 | 4.12 | 0 | 0.312 | 0.879 |
| <91 or >219 | 3 | 0.599 | 0.163 | 3.67 | 0 | 0.279 | 0.918 |
|  |  |  |  |  |  |  |  |
| Temperature |  |  |  |  |  |  |  |
| 36.1-38.0* | 0 |  |  |  |  |  |  |
| 35.1-36.0 or 38.1-39.0 | 1 | 0.261 | 0.074 | 3.5 | 0 | 0.115 | 0.407 |
| >39.0 | 2 | 0.130 | 0.133 | 0.98 | 0.325 | -0.130 | 0.390 |
| <35.1 | 3 | 0.812 | 0.233 | 3.49 | 0 | 0.356 | 1.269 |
|  |  |  |  |  |  |  |  |
| Consciousness not alert | 3 | 0.402 | 0.083 | 4.87 | 0 | 0.240 | 0.564 |
| Supplemental oxygen | 2 | 1.205 | 0.068 | 17.82 | 0 | 1.073 | 1.338 |
| Male sex | 1 | 0.318 | 0.067 | 4.77 | 0 | 0.187 | 0.448 |
|  |  |  |  |  |  |  |  |
| Age |  |  |  |  |  |  |  |
| <50* | 0 |  |  |  |  |  |  |
| 50-65 | 2 | 0.718 | 0.121 | 5.94 | 0 | 0.481 | 0.955 |
| 66-80 | 3 | 0.792 | 0.123 | 6.45 | 0 | 0.551 | 1.033 |
| >80 | 4 | 0.916 | 0.130 | 7.06 | 0 | 0.662 | 1.171 |
|  |  |  |  |  |  |  |  |
| Performance status |  |  |  |  |  |  |  |
| Level 1* | 0 |  |  |  |  |  |  |
| Level 2 | 1 | -0.071 | 0.113 | -0.63 | 0.526 | -0.292 | 0.149 |
| Level 3 | 2 | 0.175 | 0.103 | 1.69 | 0.09 | -0.027 | 0.377 |
| Level 4 | 3 | 0.402 | 0.104 | 3.86 | 0 | 0.198 | 0.607 |
| Level 5 | 4 | 0.615 | 0.123 | 5 | 0 | 0.374 | 0.856 |
|  |  |  |  |  |  |  |  |
| Renal impairment | 1 | 0.333 | 0.100 | 3.32 | 0.001 | 0.137 | 0.530 |
| Respiratory distress | 1 | 0.806 | 0.152 | 5.29 | 0 | 0.507 | 1.105 |
| Constant |  | -3.798 | 0.126 | -30.19 | 0 | -4.045 | -3.551 |

C-statistic 0.82 (95% CI 0.81 to 0.83)

*Reference category
