# Supplementary material for: Derivation and validation of a clinical severity score for acutely ill adults with suspected COVID-19: The PRIEST observational cohort study
Source: PLoS One. 2021 Jan 22;16(1):e0245840. doi: 10.1371/journal.pone.0245840 (PMC7822515; doi:10.1371/journal.pone.0245840)
Supplement: S5 Table — (DOCX) [file pone.0245840.s009.docx]

### S5 Table: Logistic regression model based on selected categorised predictor variables, excluding respiratory distress and history of renal impairment

| Predictor | Score allocated | Coefficient | SE | z | P-value | Upper 95% CI | Lower 95% CI |
| --- | --- | --- | --- | --- | --- | --- | --- |
| Respiratory rate |  |  |  |  |  |  |  |
| 12-20* | 0 |  |  |  |  |  |  |
| 9-11 | 1 | 1.543 | 1.049 | 1.47 | 0.141 | -0.512 | 3.599 |
| 21-24 | 2 | 0.334 | 0.089 | 3.74 | 0 | 0.159 | 0.508 |
| <9 or >24 | 3 | 0.733 | 0.082 | 8.98 | 0 | 0.573 | 0.892 |
|  |  |  |  |  |  |  |  |
| Oxygen saturation |  |  |  |  |  |  |  |
| >95%* | 0 |  |  |  |  |  |  |
| 94-95% | 1 | 0.355 | 0.089 | 3.96 | 0 | 0.179 | 0.530 |
| 92-93% | 2 | 0.676 | 0.111 | 6.08 | 0 | 0.458 | 0.894 |
| <92% | 3 | 1.149 | 0.084 | 13.62 | 0 | 0.984 | 1.314 |
|  |  |  |  |  |  |  |  |
| Heart rate |  |  |  |  |  |  |  |
| 51-90* | 0 |  |  |  |  |  |  |
| 41-50 or 91-110 | 1 | -0.063 | 0.077 | -0.82 | 0.415 | -0.213 | 0.088 |
| 111-130 | 2 | 0.132 | 0.096 | 1.38 | 0.168 | -0.056 | 0.319 |
| <41 or >130 | 3 | 0.335 | 0.139 | 2.41 | 0.016 | 0.062 | 0.608 |
|  |  |  |  |  |  |  |  |
| Systolic BP |  |  |  |  |  |  |  |
| 111-219* | 0 |  |  |  |  |  |  |
| 101-110 | 1 | 0.220 | 0.108 | 2.04 | 0.042 | 0.008 | 0.433 |
| 91-100 | 2 | 0.615 | 0.144 | 4.28 | 0 | 0.333 | 0.896 |
| <91 or >219 | 3 | 0.618 | 0.162 | 3.82 | 0 | 0.300 | 0.935 |
|  |  |  |  |  |  |  |  |
| Temperature |  |  |  |  |  |  |  |
| 36.1-38.0* | 0 |  |  |  |  |  |  |
| 35.1-36.0 or 38.1-39.0 | 1 | 0.259 | 0.074 | 3.49 | 0 | 0.113 | 0.404 |
| >39.0 | 2 | 0.140 | 0.132 | 1.06 | 0.289 | -0.118 | 0.398 |
| <35.1 | 3 | 0.876 | 0.229 | 3.82 | 0 | 0.427 | 1.325 |
|  |  |  |  |  |  |  |  |
| Consciousness not alert | 3 | 0.397 | 0.082 | 4.84 | 0 | 0.236 | 0.558 |
| Supplemental oxygen | 2 | 1.232 | 0.067 | 18.34 | 0 | 1.100 | 1.363 |
| Male sex | 1 | 0.320 | 0.066 | 4.83 | 0 | 0.190 | 0.450 |
|  |  |  |  |  |  |  |  |
| Age |  |  |  |  |  |  |  |
| <50* | 0 |  |  |  |  |  |  |
| 50-65 | 2 | 0.734 | 0.120 | 6.11 | 0 | 0.498 | 0.969 |
| 66-80 | 3 | 0.813 | 0.122 | 6.66 | 0 | 0.574 | 1.053 |
| >80 | 4 | 0.939 | 0.129 | 7.3 | 0 | 0.687 | 1.191 |
|  |  |  |  |  |  |  |  |
| Performance status |  |  |  |  |  |  |  |
| Level 1* | 0 |  |  |  |  |  |  |
| Level 2 | 1 | -0.057 | 0.112 | -0.51 | 0.612 | -0.276 | 0.163 |
| Level 3 | 2 | 0.212 | 0.102 | 2.08 | 0.038 | 0.012 | 0.411 |
| Level 4 | 3 | 0.437 | 0.104 | 4.22 | 0 | 0.234 | 0.640 |
| Level 5 | 4 | 0.660 | 0.122 | 5.4 | 0 | 0.420 | 0.899 |
|  |  |  |  |  |  |  |  |
| Constant |  | -3.792 | 0.125 | -30.31 | 0 | -4.037 | -3.547 |

C-statistic 0.82 (95% CI 0.81 to 0.83)

*Reference category
