# Supplementary material for: Derivation and validation of a clinical severity score for acutely ill adults with suspected COVID-19: The PRIEST observational cohort study
Source: PLoS One. 2021 Jan 22;16(1):e0245840. doi: 10.1371/journal.pone.0245840 (PMC7822515; doi:10.1371/journal.pone.0245840)
Supplement: S7 Table — (DOCX) [file pone.0245840.s011.docx]

### S7 Table: Sensitivity, specificity, PPV, NPV at each score threshold for predicting the secondary outcome of death without organ support, validation cohort

| Score threshold | Sensitivity (95% CI) | Specificity (95% CI) | Positive predictive value (95% CI) | Negative predictive value (95% CI) |
| --- | --- | --- | --- | --- |
| >0 | 1.00 (1.00, 1.00) | 0.03 (0.03, 0.03) | 0.13 (0.12, 0.13) | 1.00 (0.99, 1.00) |
| >1 | 1.00 (1.00, 1.00) | 0.09 (0.08, 0.09) | 0.13 (0.13, 0.13) | 1.00 (1.00, 1.00) |
| >2 | 1.00 (1.00, 1.00) | 0.15 (0.15, 0.16) | 0.14 (0.14, 0.14) | 1.00 (1.00, 1.00) |
| >3 | 1.00 (0.99, 1.00) | 0.23 (0.23, 0.23) | 0.15 (0.15, 0.16) | 1.00 (1.00, 1.00) |
| >4 | 0.99 (0.99, 1.00) | 0.30 (0.30, 0.31) | 0.17 (0.16, 0.17) | 1.00 (1.00, 1.00) |
| >5 | 0.98 (0.97, 0.98) | 0.38 (0.38, 0.39) | 0.18 (0.18, 0.18) | 0.99 (0.99, 0.99) |
| >6 | 0.96 (0.96, 0.96) | 0.46 (0.45, 0.46) | 0.20 (0.19, 0.20) | 0.99 (0.99, 0.99) |
| >7 | 0.93 (0.93, 0.94) | 0.53 (0.53, 0.53) | 0.22 (0.21, 0.22) | 0.98 (0.98, 0.98) |
| >8 | 0.89 (0.88, 0.90) | 0.60 (0.60, 0.61) | 0.24 (0.23, 0.24) | 0.98 (0.97, 0.98) |
| >9 | 0.84 (0.83, 0.85) | 0.67 (0.66, 0.67) | 0.26 (0.25, 0.26) | 0.97 (0.97, 0.97) |
| >10 | 0.77 (0.76, 0.78) | 0.73 (0.73, 0.74) | 0.29 (0.28, 0.29) | 0.96 (0.96, 0.96) |
| >11 | 0.70 (0.69, 0.71) | 0.79 (0.78, 0.79) | 0.31 (0.31, 0.32) | 0.95 (0.95, 0.95) |
| >12 | 0.59 (0.58, 0.60) | 0.84 (0.84, 0.85) | 0.34 (0.34, 0.35) | 0.94 (0.93, 0.94) |
| >13 | 0.50 (0.49, 0.51) | 0.89 (0.88, 0.89) | 0.38 (0.37, 0.39) | 0.93 (0.92, 0.93) |
| >14 | 0.40 (0.39, 0.41) | 0.92 (0.92, 0.92) | 0.42 (0.41, 0.43) | 0.92 (0.92, 0.92) |
| >15 | 0.32 (0.31, 0.33) | 0.95 (0.94, 0.95) | 0.45 (0.44, 0.46) | 0.91 (0.91, 0.91) |
| >16 | 0.25 (0.25, 0.26) | 0.96 (0.96, 0.97) | 0.49 (0.48, 0.51) | 0.90 (0.90, 0.91) |
| >17 | 0.17 (0.17, 0.18) | 0.98 (0.98, 0.98) | 0.52 (0.50, 0.54) | 0.89 (0.89, 0.90) |
| >18 | 0.11 (0.11, 0.12) | 0.99 (0.98, 0.99) | 0.52 (0.49, 0.54) | 0.89 (0.89, 0.89) |
| >19 | 0.08 (0.07, 0.08) | 0.99 (0.99, 0.99) | 0.60 (0.57, 0.63) | 0.89 (0.88, 0.89) |
| >20 | 0.05 (0.04, 0.05) | 1.00 (1.00, 1.00) | 0.63 (0.59, 0.67) | 0.88 (0.88, 0.89) |
| >21 | 0.03 (0.03, 0.04) | 1.00 (1.00, 1.00) | 0.72 (0.66, 0.76) | 0.88 (0.88, 0.88) |
| >22 | 0.02 (0.01, 0.02) | 1.00 (1.00, 1.00) | 0.76 (0.68, 0.83) | 0.88 (0.88, 0.88) |
| >23 | 0.01 (0.01, 0.01) | 1.00 (1.00, 1.00) | 0.80 (0.68, 0.88) | 0.88 (0.88, 0.88) |
| >24 | 0.01 (0.00, 0.01) | 1.00 (1.00, 1.00) | 0.86 (0.66, 0.96) | 0.88 (0.88, 0.88) |
