# Supplementary material for: Derivation and validation of a clinical severity score for acutely ill adults with suspected COVID-19: The PRIEST observational cohort study
Source: PLoS One. 2021 Jan 22;16(1):e0245840. doi: 10.1371/journal.pone.0245840 (PMC7822515; doi:10.1371/journal.pone.0245840)
Supplement: S3 Appendix — (DOCX) [file pone.0245840.s014.docx]

## S3 Appendix: The National Early Warning Score (version 2)

NEWS2 is the latest version of the National Early Warning Score that the Royal College of Physicians (London) advocates as a system to standardise the assessment and response to acute illness (see <https://www.rcplondon.ac.uk/projects/outputs/national-early-warning-score-news-2>). It has seven parameters, each of which are scored from zero to three providing an overall score between zero and 20. The scores for each parameter are shown in the table below. The scale for patients with confirmed hypercapnic respiratory failure was not used in our analysis.

| Score | 3 | 2 | 1 | 0 | 1 | 2 | 3 |
| --- | --- | --- | --- | --- | --- | --- | --- |
| Respiratory Rate | ≤8 |  | 9-11 | 12-20 |  | 21-24 | ≥25 |
| Oxygen saturation | ≤91 | 92-93 | 94-95 | ≥96 |  |  |  |
| Heart Rate | ≤40 |  | 41-50 | 51-90 | 91-110 | 111-130 | ≥131 |
| Systolic BP | ≤90 | 91-100 | 101-110 | 111-219 |  |  | ≥220 |
| Temperature | ≤35.0 |  | 35.1-36.0 | 36.1-38.0 | 38.1-39.0 | ≥39.1 |  |
| Neurology |  |  |  | Alert |  |  | Confusion, Voice, Pain, Unresponsive |
| Air or Oxygen |  | Oxygen (based on FiO_2_>21%, or FiO_2_>0 L/min) |  | Air |  |  |  |
