# Supplementary material for: Derivation and validation of a clinical severity score for acutely ill adults with suspected COVID-19: The PRIEST observational cohort study
Source: PLoS One. 2021 Jan 22;16(1):e0245840. doi: 10.1371/journal.pone.0245840 (PMC7822515; doi:10.1371/journal.pone.0245840)
Supplement: Attachment — Submitted filename: Response to review 1.docx [file pone.0245840.s018.docx]

Thank you for considering our paper and providing the reviewer’s thoughtful comments. We have addressed them in a revised version of our paper. Our responses to each specific comment are as follows:

Editor:

*I do wish to emphasize one point, the authors need to emphasize their uniqueness among various other scores recently published.*

RESPONSE: We have extensively rewritten the discussion to address this point. The second paragraph has been added to describe the methodological features, including the study population selection criteria and choice of outcome, that ensure we have developed an optimal score. The third paragraph describes how our score improves upon previously recommended scores, specifically NEWS2 and PMEWS. We have rewritten paragraph five, critiquing other prediction scores, and we have added to paragraph six on rapid clinical scores to highlight their limitations compared to ours.

Reviewer #1:

*1. The patient data was derived during the first wave of COVID-19 in the UK. The second wave in the UK, and elsewhere, appears to have some different characteristics. Could the authors comment on this and / or speculate on the need to repeat their data collection in other countries / during the current (second) wave, to establish whether it c statistic is affected?*

RESPONSE: We have added a sentence to the last paragraph of the discussion suggesting this as a future research priority.

*2. Might the authors data have a significant selection bias in that the location of data collection was emergency departments? Patients will have presented to primary care, Acute Medical Units etc etc hence how certain can we be that this result is generalisable?*

RESPONSE: We agree that our data are most applicable to patients presenting to the ED and less applicable to patients presenting elsewhere. We have added consideration of this to the last paragraph of the discussion, along with a research recommendation.

*3. Do the authors have any data to suggest what the short-term outcome of their patients was - e.g. how many were receiving level 1, level 2 and level 3 care at 72 hours post presentation? If not, might this secondary outcome be useful in future studies?*

RESPONSE: We have added initial location of admission (ICU, HDU or ward) to Table 1.

*4. Could the authors please include the data for patient sex in Table 2?*

RESPONSE: We have added patient sex to Table 2.

*5. Could the authors comment on the fact that ~67% of patients were admitted but only 31% were SARS-CoV-2 positive?*

RESPONSE: We have added a sentence to the methods clarify that only admitted patients were tested at the participating sites during the first wave and noting in the results that the 31% positivity rate reflects a combination of lack of testing in those discharged, suboptimal sensitivity of standard tests, and the difficulty of differentiating COVID-19 from similar presentations. We feel this represents an expected rate for a clinically relevant cohort with suspected rather than confirmed COVID-19 (see response to reviewer #2, Q1).

*6. Could the authors comment further on the very high proportion of missing data related to supplemental oxygen, and the much larger proportion in the validation cohort?*

RESPONSE: We recognised that missing data could be a problem, given that oxygen supplementation is often poorly recorded in clinical notes, and planned multiple analyses using different approaches to handling missing data. We identify this as a limitation in the discussion – “Recording of inspired oxygen concentration was subject to a particularly high rate of missing data. We anticipated this problem and pre-specified analyses involving multiple imputation, deterministic imputation, and complete case analysis to explore the impact of missing data. There was reasonable concordance between the models”. We randomly allocated sites to the derivation and validation cohorts, so the higher rate of missing data in the validation cohort reflects random allocation of sites with higher missing data to the validation cohort.

*7. Could the authors please provide more detail in figure 3, in particular, the number of patients with each score and the probabilities for each score 17-29?*

RESPONSE: We have added the number of patients with each score and the probabilities for each score 17-29.

*8. Given that the authors cut off for risk of a "bad outcome" is stated as a score >4 / probability of >9%, which can be achieved by being a 50 year old male with a temperature of 38.1 and a heart rate of 91 seems poorly calibrated to real life?*

RESPONSE: We have added to paragraph eight of the discussion to address this important point. The key issue is that the score was developed upon, and therefore applies to, people who have attended the ED with suspected COVID-19. A patient with the characteristics described who did not need to attend the ED would probably not have a high risk of adverse outcome. However, our findings show that in patients attending the ED with a high baseline risks even people with a little or no physiological abnormality have a significant risk of adverse outcome. This does not necessarily mean they have to be admitted, but they should not be discharged with false reassurance that they are at low risk.

*9. As currently written, I am unconvinced that the authors addition of age, sex and performance status adds anything useful to NEWS2 as a triage tool, especially in the absence of knowing whether the patient is positive for SARS-CoV-2. Perhaps they could clarify their justification for adding these variables to their real-world, pre-diagnosis target population?*

RESPONSE: We have added the third paragraph of the discussion to address this point. Adding these variables improves the prediction, as measured by the c-statistic. The practical impact of this is to ensure that pre-morbid risk factors are taken into account when using NEWS2. Thus, a young person with unrestricted performance status is identified as being at low risk of adverse outcome, even if their NEWS2 score is 3-4, whereas an older person is identified as being at higher risk of adverse outcome, even if their NEWS2 score is 0-2. Decision-making on the basis of NEWS2 alone is likely to result in over-triage of younger, healthy people to unnecessary admission, and under-triage of older people with restricted performance status to inappropriate discharge.

Reviewer #2:

*The study is well-written and easy to read. Methods are well described and explained and the statistical analysis is appropriate. Results are interesting and the score could be useful in clinical practice in the event of a third pandemic wave. The two main strengths of the study are the large sample size and the appropriate method for building the score. One of the main limitations is the added-value of this new score, compared to existing triage scores for patients admitted to emergency department. I have some additional concerns that need to be discussed.*

RESPONSE: We have extensively rewritten the discussion, with the addition of paragraphs two and three, to describe the added value of the new score.

*1. Why did you consider patients with suspected and not confirmed COVID-19? In fact, this score could be used for all patients admitted to the emergency department, regardless of their COVID-19 status and is therefore not very different from existing triage scores. It would have been more interesting to develop a specific score for COVID-19 patients. Please clarify this point. In this regard, it would be interesting to repeat the analysis only in patients with confirmed COVID-19 if you are convinced of the need to develop specific predictive scores for COVID-19 patients.*

RESPONSE: We have added to the first paragraph of the introduction to explain why we selected those with suspected COVID-19 and added to the second paragraph of the discussion why we feel this is a strength of our study. ED triage tools need to be used prospectively, when the diagnosis of COVID-19 is still only suspected in most cases, rather than retrospectively, when it has been confirmed. Rapid tests may be used to confirm COVID-19 in the ED, but limited sensitivity means COVID-19 cannot be ruled out in cases with a strong clinical suspicion, so COVID-19 still needs to be suspected in the absence of positive testing. Furthermore, despite much talk of a roll-out of rapid tests, we are now well into the second wave of the pandemic and use of rapid tests remains limited.

It would be interesting to repeat the analysis only in those with confirmed COVID-19 but the lack of testing on those discharged would mean that this effectively limits the cohort to those admitted, thus incurring the issues associated with limiting the cohort to patients whose selection for admission is likely to be based on key predictor variables.

*2. What was the proportion of patients in whom COVID-19 was confirmed? This is a very important point before discussing your results, since the rationale for your study is based on the need to develop scores for COVID-19. Your results cannot be considered in the same way according to the proportion of confirmed COVID-19 patients.*

RESPONSE: Table 1 shows that COVID-19 was confirmed in 31.1% of the derivation cohort and 31.4% of the validation cohort. We have added a sentence to the methods to clarify that only admitted patients were tested and noting in the results that the 31% positivity rate reflects a combination of lack of testing in those discharged, suboptimal sensitivity of standard tests, and the difficulty of differentiating COVID-19 from similar presentations. We feel this is an expected rate for a clinically-relevant cohort with suspected rather than confirmed COVID-19.

*3. You state in the opening of the discussion that you included the NEWS2 score in your own score in addition to age, sex and performance status. However, you never described the NEWS2 score in the manuscript and in the Figure 1 (which is a Table), which is very confusing for readers. From my point of view, it would be clearer to simply indicate all the variable you included in your score rather than talking about the News2 score. Please clearly explain what the NEWS2 score is and clarify this point.*

RESPONSE: We have added Appendix 3 to describe the NEWS2 score.

*4. In figure 2, since your score ranged from 0 to 29, why did you censor the data after a score of 16 by merging all scores > 17? Please also indicate the probability of adverse outcomes for each score > 17.*

RESPONSE: We collapsed scores above 17 into one column because the numbers in each strata above 17 were relatively small, and thus subject to greater random variation. We have amended the figure to include separate scores above 17.

*5. It is of importance to provide in the manuscript a table and/or a figure summarizing the key findings of the first part of the study (derivation cohort).*

RESPONSE: We have added a new figure (Figure 1) summarising the derivation part of the study and have renumbered subsequent figures.

*6. Please further discuss your score in the light of the existing literature, not only by considering its ability to predict adverse outcomes (c-statistic) but also by considering its potential added-value (relevance of variables, ease of use…). In addition, the ability of your score to predict adverse outcomes should be also further discussed especially the sensitivity and specificity you found.*

RESPONSE: We have added paragraph three of the discussion and extended paragraph five to discuss our score in the light of existing literature and consider its potential added value, and have extended paragraph eight to discuss how it could be used to predict adverse outcome.

*7. It would have been very interesting to compare your score to the results of the PAINTED study and to the scores developed for influenza pandemic. Please discuss this specific point.*

RESPONSE: We have added paragraph three of the discussion to address this point. We used data from our previous analysis of existing scores in the PRIEST data to compare our score to those developed for the influenza pandemic, since this provides much greater precision than the PAINTED data and a direct comparison using the same cohort.
