# Supplementary material for: Derivation and validation of a clinical severity score for acutely ill adults with suspected COVID-19: The PRIEST observational cohort study
Source: PLoS One. 2021 Jan 22;16(1):e0245840. doi: 10.1371/journal.pone.0245840 (PMC7822515; doi:10.1371/journal.pone.0245840)
Supplement: Attachment — Submitted filename: Response to review 2.docx [file pone.0245840.s019.docx]

Thank you for considering our paper and providing the additional reviewer comments. We have revised the paper to address them and outline our responses to each point below.

Reviewer #1: Thank you for considering the points raised by both reviewers and making related changes to the manuscript.

I have the following residual concerns.

1. The primary outcome has been chosen for pragmatic reasons however predicting death or the need for organ support in the 30 days following presentation to ED ignores at least 2 very important groups, namely those who were admitted but did not required organ support, and those that did not receive organ support but did die after 30 days.

RESPONSE: We have added this to the limitations section of the discussion.

2. A minor point related to the penultimate paragraph on page 6, please include the total number of deaths observed - all that is stated is that the number was >200 [being 1% of the 20,000 used in the undocumented power calculation].

RESPONSE: We have added the adverse outcome rate in our cohort to this paragraph (not the death rate, since the sample size estimate was based on 200 adverse events, rather than deaths).

3. The proportion of missing data and the large difference between the derivation and validation cohorts in the "air or supplemental oxygen" question are problematic.

RESPONSE: We acknowledge in the discussion that the high rate of missing data for "air or supplemental oxygen" is an important limitation and describe the approaches we took to handling missing data. We randomly allocated sites to derivation and validation cohorts, so the difference between the cohorts is unlikely to be systematic.

4. The first sentence of the first paragraph in the discussion is missing several vital caveats as follows:

"We have developed a clinical illness severity score for acutely ill patients, WHO PRESENTED TO EMERGENCY DEPARTMENTS IN THE UK BETWEEN MARCH AND JUNE[??] with suspected COVID-19" the clinical context, geography and calendar are critical.

" . . . to predict the risk of death or receipt of organ support IN THE FOLLOWING 30 DAYS"

". . . predicting adverse outcome with A high sensitivity BUT A VERY LOW SPECIFICITY."

RESPONSE: We have added these caveats, with the exception of the location and timing of data collection. Science inevitably involves generalizing findings beyond the analysed data. In the discussion we consider potential limitations on generalizability and the need to validate in other settings and waves of the pandemic.

5. The authors appear to be simultaneously proposing their score be used to inform triage decisions in ED with a clear bias towards overtriage i.e. admitting some patients who at very low risk of the adverse outcome they define; and yet give a clinical example in which "patient preference" rather than clinical judgement should be a relevant factor. The latter assumes that the triage decision is not being made in the clinical context of a stressed / overwhelmed hospital system.

RESPONSE: We address this issue in the discussion and have added availability of resources to the factors that need to be considered alongside risk of adverse outcome in clinical decision-making.

6. Similarly, the authors make the case that their score is superior to certain others because it includes acute physiology plus age and performance status but seem to believe that clinicians do not take these chronic factors into account when using acute scores to assist in objectively grading severity of illness. Furthermore, do the authors really believe that their c-statistic of 0.80 is clinically rather than statistically better than the 0.75 and 0.77 for CURB-65 and NEWS2?

RESPONSE: We believe that it is up to clinicians how they use our score, or any alternative, to predict adverse outcome and support decision-making. Our analysis presents estimates of prognostic accuracy to inform clinicians in making their choices. We believe that a c-statistic of 0.80 is clinically significantly better than a c-statistic of 0.75 or 0.77, but our belief is not important. What is important is that we have presented our findings in a transparent manner that allows readers to judge for themselves whether the additional complexity of our score compared to NEWS2 or CURB-65 is worth the improved prognostic value.

7. The exclusion of any discriminating laboratory / diagnostic tests from a population who have attended a hospital appears to be an effort to provide a means of turning patients away at first triage. The authors have not demonstrated that the addition of any such variables fails to improve the discrimination, especially the specificity of their score. Given that accurate and rapid point-of-care testing is now widely available, is turnaround time in ED too critical to have considered the potential value of such tests?

RESPONSE: We have described the reasons why we think that a clinical score (without laboratory or radiological tests) is most useful and of greatest interest to clinicians working in the emergency department. This is why we have made this the principal output of our analysis. We have data available to explore whether laboratory and radiological information can improve prediction, but for the reasons outlined in our paper, we have not made this a priority in our plans for analysis and dissemination.

8. The authors should comment on the QCOVID score - https://pubmed.ncbi.nlm.nih.gov/33082154/ - and consider the accompanying editorial.

RESPONSE: The QCOVID score was developed in a population cohort study to predict the risk of death from COVID-19 in the general population. It is clearly very useful for guiding public health interventions, such as shielding for high-risk individuals and targeting vaccination, but has a very different purpose to our score, which is aimed at predicting the risk of adverse outcome among people who are acutely ill with COVID-19.

Reviewer #2: Dear authors, you have taken into account all my comments and suggestions and provided a detailed point-by-point response. The manuscript has been significantly improved and I have no additional comments. Best regards.

RESPONSE: Thank you
